# Enhancing Transparency, Auditability, and Reproducibility of Deduplication in Systematic Reviews: Tutorial for the Rayyan Method and Systematic Auto Resolver

**DOI:** 10.2196/94317

**Published:** 2026-07-14

**Authors:** Mimi M Kim, Amin M Shanaa, Hossam Hammady, Mohammad Aboelnour, Robert Ayan

**Affiliations:** 1 Rayyan Systems Inc., 1 Broadway, 14th Floor, Cambridge, MA, 02142, United States, 1 617 453-2567

**Keywords:** systematic review methodology, deduplication, software demonstration, transparency, evidence synthesis

## Abstract

Deduplication across search results is one of the earliest and most critical steps in the systematic review methodological process; yet, existing solutions often lack the transparency, auditability, and reproducibility required by rigorous systematic review standards. Many automated deduplication tools introduce bias through opaque, nonconfigurable algorithmic decisions, while also potentially removing relevant references through false positive identification. We provide a tutorial on the Rayyan Method, including the Systematic Auto Resolver feature for the deduplication process. This method is defined by an enhanced deduplication approach that combines high-sensitivity duplicate detection with user-controlled resolution criteria. Systematic Auto Resolver enables research teams to define, apply, and document their own deduplication standards rather than relying on predetermined automated decisions. Users apply deduplication criteria, iteratively reviewing results after each pass, maintaining complete control over methodological decisions. The Rayyan Method addresses critical limitations in current deduplication approaches by supporting methodological rigor through user-controlled resolution while enhancing efficiency, transparency, and reproducibility. By empowering research teams to define their own deduplication criteria, this approach supports and aligns with the prescribed methodologically rigorous systematic review process. The method provides a citable framework for researchers to comprehensively document their deduplication methodology.

## Introduction

The escalating demand for evidence-based decision-making across diverse sectors, notably health care and policy, has amplified the reliance on methodologically rigorous systematic reviews for robust evidence synthesis [1]. The systematic review process begins with a carefully constructed literature search strategy, adhering to predefined, transparent, and registered criteria [2]. Central to maintaining the rigor of systematic reviews is the effective deduplication of search results, a crucial step to minimize reviewer workload, mitigate bias, and ensure the accuracy of findings [3].

While many software platforms offer a deduplication feature, their functionalities can lack the full integration, auditability, transparency, and reproducibility that are essential for regulatory applications [4,5]. With the increasing automation of research workflows, concerns have grown commensurately about the critical need to maintain controlled, transparent processes in regulatory contexts, particularly with the application of artificial intelligence. However, many researchers have adopted deduplication solutions that may inadvertently remove or cause the removal of relevant evidence in pursuit of identifying the most complete set of duplicates. This is inherently contradictory and perilous to the methodology.

## The Multidimensional Challenge of Defining Duplicate References

The primary challenge of this stage of the review stems from the absence of a consistent definition of what constitutes a duplicate in an evidence synthesis context. For example, duplicates have been defined as “the same article published in the same place, while the same article published in a different place is not a duplicate” [6]. In contrast, duplicate records have been described as “being the same bibliographic record (irrespective of how the citation details were reported; eg, variations in page numbers, author details, accents used, or abridged titles)” [7,8]. Additional factors that may result in duplicate records include intradatabase and interdatabase duplication due to differences in publication year, volume, or page numbers resulting from early release or online-first publication [9]. These examples of varied definitions highlight the need for deduplication solutions to address the primary challenge by accommodating the varying criteria used to define duplicate references.

A critical dichotomy has emerged with the increasing number of available solutions: (1) the failure to detect duplicates with single-character deviations or greater (where missing, incomplete, and erroneous data cause duplicates to be missed entirely) and (2) the failure to avoid false positives (where highly similar references are erroneously resolved as duplicates). The result can be significantly impactful when the removal of relevant evidence identified as false positives compromises the integrity of systematic reviews. This can raise concerns about biased conclusions stemming from omitted evidence, where any research produced using such a tool would be suspected of the same. Consequently, there is a critical need for testing, auditing, and validation of deduplication methods and solutions. When solutions offer a single method of deduplication, the method, concerningly, minimizes human expertise in the method’s application [7,10].

Rayyan’s approach is designed to address this duplication complexity by fulfilling 2 research paradigms. First, convenience, where researchers rely on automated tools for deduplication while recognizing the dependency on the applied method or algorithm and the data assessed. Second, integrity, where researchers follow transparent, auditable, and reproducible procedures to ensure methodological rigor when removing inexact duplicates.

## History and Evolution

Rayyan was originally conceived with the objective of supporting the most time-consuming aspects of systematic literature reviews by enhancing the process of deduplication and screening of titles and abstracts for researchers with varying experience and expertise across a broad spectrum of review types (eg, literature reviews, scoping reviews, and systematic reviews). Initially, Rayyan offered automatic duplicate detection; however, after realizing that automatic detection violated the platform’s mission to protect full user control, Rayyan updated the design to execute a semiautomated process with a foundational commitment to a level of usability governed by speed, accuracy, and simplicity while maintaining full user control.

We, as the Rayyan team, aimed to design a comprehensive systematic review application enabling human-computer research collaboration where research teams have a solution that conforms to their unique requirements, methods, standards, and even individual preferences. Rayyan’s user-centric design philosophy is driven by our commitment to methodological rigor without imposing a single *one-size-fits-all* approach to systematic reviews, including the important and sensitive challenge of deduplication. Further details on the history of Rayyan’s inception, architecture, usability, accuracy against manual methods, and the added value of Rayyan’s other features are published elsewhere [10].

## Objective

This tutorial specifically describes the deduplication process using the unique capabilities of Systematic Auto Resolver and its advantages. Together, the capabilities of Rayyan combined with the user-defined deduplication criteria constitute a deduplication method, henceforth referred to as the Rayyan Method.

## The Rayyan Method

### Background

The Rayyan Method is guided by the principle of implementing human control and oversight rather than a human-in-the-loop paradigm. In human-in-the-loop systems, humans are often informed of the results after automated actions occur [11]. The Rayyan Method instead starts with human initiation and guidance of deduplication decisions, preventing the automated removal of potentially relevant evidence. This distinction ensures that humans initiate the action and guide it with human expertise.

The pioneering work of methodologist Bramer established the concept of a manual series of deduplication criteria to achieve superior deduplication results beyond exact duplicates [4]. The Rayyan Method also meets the same objectives and extends the process by providing researchers with the capability where deduplication criteria are defined by the user, rather than predetermined by software developers. This evolution represents a shift from fixed procedural steps to adaptable, user-controlled criteria that can accommodate the diversity of research contexts and requirements.

Rayyan offers an advanced duplicate detection algorithm within both its web-based and mobile app designed for the systematic review process [10]. Using greater sensitivity, Rayyan detects more possible duplicates than other solutions [7]. Using precision rules, Rayyan minimizes false positives and false negatives by allowing the review team to specify which deduplication criteria to apply to their dataset. The applied deduplication criteria can be reported for transparent, auditable, and reproducible reporting of the deduplication method. Any possible duplicates that are not removed by the application of the deduplication criteria can be manually reviewed and resolved. Manual resolution is most common for instances of multiplicity stemming from high similarity, where the research team is then able to decide whether to keep one or both references.

**Figure 1.**
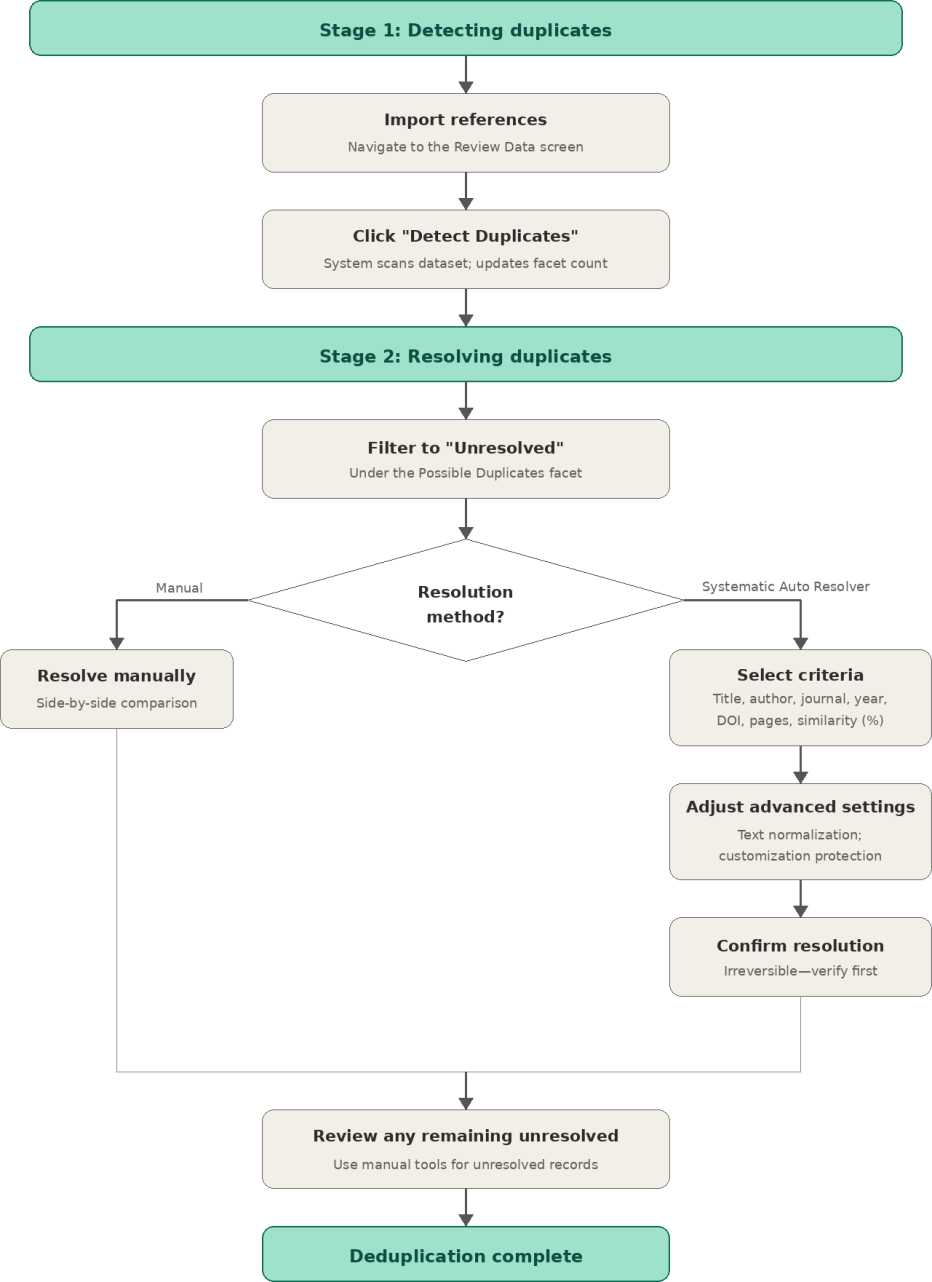
Deduplication and Systematic Auto Resolver step-by-step process.

This is a 2-stage process that separates detection from resolution (Figure 1). This separation is critical because it allows for maximum sensitivity in detection without the risk of false positives in resolution. In stage 1, Rayyan casts a wide net to identify all possible duplicates to delegate precision to stage 2 resolution. In stage 2, research teams apply their own criteria to resolve these duplicates systematically and transparently.

### Stage 1: Detecting Duplicates

Research teams start by importing all their search files containing the references that constitute the possible evidence base for their systematic review into the Rayyan platform. Rayyan also offers a *PDF first* import option where the platform creates the references and retrieves metadata based on the initial PDF upload. Rayyan currently supports importing 1 GB for ZIP files, 100 MB for reference files, and 10 MB for PDFs, with support for a large array of industry-standard content formats (EndNote, RefMan, BibTeX, PubMed, Web of Science, PDF, and more) and file formats (.enw, .ris, .bib, .csv, .wos, .txt, .xml, .doc, .zip, and .pdf). The simultaneous import of more than 10 search files may be repeated any number of times to add additional references to a review for processing in real time. Any limitation on the size of the search files, the number of search files that may be simultaneously imported, or the number of references that duplicate detection can handle has been specified as a safeguard to prevent misuse of the platform, given that duplicate detection is a free feature of Rayyan available to anyone. However, users may also overcome the 10-file limit by importing searches from Rayyan’s “My Library” to a review.

Once all references are uploaded to Rayyan by the review team, the deduplication process is user initiated with one click of the Detect Duplicates button, returning a set of records identified as “Possible Duplicates” as a filtered index of records. The Detect Duplicates button may be found in several locations, including the Overview page, Review Data page, or the Rayyan mobile app. Initiating the duplicate detection process from any of these interfaces or via the application programming interface has the same effect. Duplicate detection and removal are user initiated, meaning deduplication is neither automatic nor required to use Rayyan to complete an end-to-end systematic review workflow.

Rayyan’s detection algorithm identifies similar records [10] to generate “Possible Duplicates,” defined as having sufficient similarity to other records. The returned results include a set of possible duplicate records with their individual similarity scores presented in an additional column. In independent third-party evaluations, Rayyan possessed remarkably high and consistent accuracy, sensitivity, and specificity for identifying duplicate references [7,12].

### Step 2: Resolving Duplicates

#### Selecting Deduplication Criteria

After executing Detect Duplicates, the end user is provided with a similarity score (percentage of similarity) for records sharing similarities. The end user is presented with the option to manually resolve or autoresolve these potential duplicates based on the criteria defined a priori. The Systematic Auto Resolver empowers the end user with control over the criteria used to define duplicates within the set of detected possible duplicates. For example, a review team might choose title, author, year of publication, and DOI to ensure accuracy. Once the universe of possible duplicates has been identified, the process of resolving duplicates manually and with the Systematic Auto Resolver is enabled.

The deduplication criteria available for selection include the following:

- Title
- Author
- Journal
- Year
- Pages
- DOI
- Publication type
- Overall similarity score (percentage of similarity based on title, journal, year, and author information)

These criteria can be applied individually or in combination. The end user specifies the deduplication criteria that are applied to resolve duplicates. This level of control allows for tailored deduplication strategies that are not possible with fully automated tools.

#### Manual Resolution of Duplicates

The first stage of duplicate resolution is manual resolution. By selecting the “Unresolved” filter under the “Possible Duplicates” filter on the “Review Data” screen, a list of possible duplicate records is shown. By selecting a record listed within the Unresolved filter and clicking “Resolve Duplicates,” paired combinations of possible duplicates, in plurality for those instances, are presented side by side for the end user to resolve manually. This manual process intends to provide maximum control and human oversight over the verification and removal of duplicates from the systematic review. The steps for manual resolution of duplicates are as follows:

1. Visit the “Review Data” screen
2. Click “Detect Duplicates” to complete duplicate detection
3. Select “Unresolved” from the Possible Duplicates facet
4. Review and resolve duplicates manually by selecting which records to keep (left, right, or both) or marking them as “Not Duplicate”

To export the deleted records, select the “Deleted” filter in the Possible Duplicates facet. Then select “Export” and select “Filtered” to export only the deleted records for reporting.

#### Systematic Auto Resolver Feature for Duplicate Resolution

The second stage of duplicate resolution is to apply Systematic Auto Resolver and is summarized in the following steps:

1. Access the Systematic Auto Resolver
2. Select the deduplication criteria
3. Adjust advanced settings
4. Confirm autoresolution
5. View notifications and results

Research teams may repeat the application of the Systematic Auto Resolver with a progressive series of rules from the most conservative to the most encompassing or resort to manual resolution to deal with unresolved possible duplicates that are resistant to the application of rules. The ability to control and exercise oversight is intended to minimize the risk of undetected false positives that can occur when single-stage automated tools delete high-similarity possible duplicates as duplicates without user authorization or visibility. It is important to note, however, that the Systematic Auto Resolver is not immune to false positives, particularly when few criteria are applied without sufficient specificity or methodological grounding. The iterative, criteria-based approach described in this tutorial is designed to reduce this risk, and the auditable record of all resolution decisions ensures that any errors remain traceable and correctable by the research team.

While concerns have heightened over the increasing automation of systematic reviews, with particular sensitivity around the behavior of artificial intelligence, many ignore the seemingly innocuous endorsement of single-stage deduplication tools that may cause the removal of relevant evidence without human control or oversight. Like other early errors in the systematic review process, such as poor protocol development, deficient search strategies, or poor search implementation, deleting relevant evidence using an automation tool jeopardizes the integrity of the systematic review process from the earliest steps. Tool adoption becomes an implicit endorsement of the deduplication method it uses. Unlike single-stage automated tools that remove duplicates without user authorization or visibility, the Rayyan Method ensures that resolution decisions, whether manual or criteria-based, are explicitly authorized, documented, and auditable by the research team. This 2-step deduplication process does not entirely eliminate the possibility of false positives but ensures that any such errors are traceable to specific user-defined criteria rather than opaque algorithmic decisions, preserving the researcher’s ability to identify, review, and correct duplicate cases.

However, user control is only as rigorous as the methodological decisions informing it. Transparency in tooling does not substitute for competency in application; instead, it transfers responsibility to the reviewer. This is a meaningful tradeoff: where closed, automated tools make implicit methodological decisions on the user’s behalf, the Rayyan Method requires users to make those decisions explicitly and deliberately. For researchers without deep familiarity with deduplication methodology, this degree of control can itself become a source of error if criteria are selected without an understanding of their relative reliability, limitations, or appropriate sequencing (eg, applying DOI as a sole matching criterion can systematically overlook duplicates in records with incomplete or “dirty” metadata). The considerations that follow are designed to address this directly by providing not only procedural steps but also the methodological rationale behind each decision point, including known field-specific limitations that reviewers should consider before proceeding. Readers seeking a broader evidence base for criteria selection are encouraged to consult the methodological literature on deduplication field performance [4,6,7,13].

### Additional Considerations

#### Options for Mitigating Risks of False Positives and False Negatives When Using Systematic Auto Resolver

Optimizing the selection of criteria requires an understanding that no single field is perfectly reliable across all records or databases [4]. There is variation across all the criterion fields in how they are populated and/or formatted, which directly impacts sensitivity (detecting more true duplicates) and specificity (avoiding the removal of unique records). The evidence base includes literature on the tradeoffs between sensitivity and specificity and should be considered when configuring a criterion approach [4,7]. We recommend selecting criteria based on published validation evidence rather than ad hoc testing. The methodological literature provides empirically grounded guidance on the relative reliability of individual matching fields [4,7,8], which reviewers should consult when configuring Systematic Auto Resolver passes. A stepwise approach, beginning with high-specificity combinations and progressively broadening, has been shown to maximize duplicate detection while minimizing the risk of false-positive removal. To inform the definition of this approach, we provide the following considerations for the various criterion fields: Title; Year, Author, and Journal; DOI; Pages and Publication Type; and Overall Similarity Percentage.

*Title* is a consistently populated and stable field across databases but also has varying representations across databases. With Rayyan, text normalization, which converts text to lowercase and removes punctuation (discussed further below), should be enabled to execute title-based matching and minimize minor formatting differences between database records that can lead to true duplicates being missed.

*Year*, *Author*, and *Journal* are criteria that can serve as confirmatory criteria used in conjunction with Title rather than as standalone matching fields. Year is a supplement that can reduce false positives from a similar titled but distinct articles, thereby reducing false positives. Author fields commonly have variations across databases (eg, full name vs initials, surname order), which can reduce recall when used alone as an identifier. Journal fields are reliable when well indexed in biomedical literature and less so for gray literature, conference proceedings, or preprints. Overall, these fields are subject to variation across databases that can significantly impact duplicate detection and resolution.

*DOI* is considered a highly precise criterion but with lower recall. DOIs that are confirmed as matched can be reliably considered true duplicates; however, DOI metadata can be limited or erroneous for a proportion of records [14,15]. Consequently, we recommend DOI as a supplementary confirmatory criterion in later iterations and not as a primary or sole matching criterion.

Additionally, *Pages and Publication Type* are also less reliable as standalone criteria. An important consideration is that the formatting of page numbers frequently varies across databases (eg, first page only vs full range; online-first vs final print pagination). The same is often the case for Publication Type. Therefore, these fields are potentially most useful as supplementary criteria within multifield combinations rather than as primary matching criteria.

Finally, as reported in the Rayyan platform, the *Overall Similarity Percentage* is potentially most useful for capturing duplicates with inconsistent metadata across multiple fields. Higher thresholds of ≥95% approximate exact matching with tolerance for minor variation and carry a lower risk of false positives. Lower thresholds increase the number of duplicates detected but require more careful manual review of flagged records. We recommend beginning at ≥95% and reviewing the results before considering a lower threshold.

#### Applying Criteria Iteratively

Rather than applying all criteria in a single pass, we recommend a stepwise approach grounded in the evidence base, as established by Bramer et al [4], among others [15]. Evidence strongly supports that applying a multistep method that begins with high-specificity field combinations and then broadens the criteria can outperform single-pass deduplication approaches [4]. With this evidence-based guidance in mind, Rayyan’s Systematic Auto Resolver is designed to support sequential passes with criteria sets. An example of a practical evidence-based methodological sequence for many review types could be as follows:

- Pass 1: Title + Year + Author. This combination captures the majority of exact and near-exact duplicates with minimal risk of false positives. It is the appropriate starting point for most datasets.
- Pass 2: Title + Journal, or Title alone with text normalization enabled. This pass targets records with author field inconsistencies or missing author metadata that may have been missed in Pass 1.
- Pass 3: Previous fields + DOI + Pages + Publication Type. A final high-confidence pass for any remaining unresolved records where additional metadata are present and verified.
- Pass 4: Overall Similarity at ≥95%. Review flagged records before confirming resolution, as this pass is most likely to surface borderline cases requiring judgment.

Rayyan does not take any action specifically not authorized by end users. This means the integrity of the results is entirely in the hands of the researcher, where the application of methodological rigor guarantees integrity. Rayyan’s role in this instance is to apply the tools as instructed by the user to produce the results. It is important to note that because Systematic Auto Resolver decisions are irreversible, prioritizing specificity in early passes and reserving broader criteria for later passes, where manual review of flagged results is feasible, reduces the risk of inadvertently removing unique records.

Tool transparency does not guarantee review transparency. Rayyan’s Systematic Auto Resolver provides a transparent and auditable process, but this only contributes to a reproducible review record if researchers actively document and report their deduplication decisions. In accordance with PRISMA (Preferred Reporting Items for Systematic Reviews and Meta-Analyses) 2020 reporting standards [1] and best practices for deduplication reporting [4,7,13], researchers should document the following elements in their methods section, registered protocol, or supplementary materials:

- Tool and date: The name of the deduplication software used and the date on which deduplication was performed. Software features and criteria may be updated over time, so the date of use contextualizes the methodology for future readers.
- Detection method: Whether duplicate detection was initiated via the Detect Duplicates function and any settings applied at that stage.
- Criteria per pass: For each Systematic Auto Resolver pass, the specific criteria applied (eg, Title + Year + Author; Overall Similarity ≥95%), the sequence of passes, and whether text normalization or customization protection was enabled.
- Resolution counts: The total number of references flagged as possible duplicates; the number resolved via Systematic Auto Resolver; the number resolved manually; and the number remaining unresolved after all passes.
- Criteria rationale: A brief statement of the reasoning behind criteria selection, particularly where choices were informed by dataset-specific factors or deviated from standard stepwise practice.

Research teams that have conducted deduplication partially or entirely outside Rayyan should note that Rayyan’s PRISMA flow diagram generator allows *n* values to be manually overridden to ensure the completed diagram accurately reflects the full deduplication process, regardless of where it was performed. This should be documented in the methods section alongside the other deduplication reporting elements listed above. Thorough documentation of these elements ensures that the deduplication process is fully citable, replicable by other research teams, and auditable by journal reviewers and editors.

### Advanced Settings

In addition, the following advanced settings may be applied as part of the deduplication criteria.

*Select Default Search*: The end user may specify which imported dataset to preserve when duplicates are detected across different searches. This setting is applied independently for each Systematic Auto Resolver pass, meaning researchers can select a different default dataset across passes to reflect the relative quality or completeness of metadata from different sources for a given criteria set. For any duplicates that remain unresolved after Systematic Auto Resolver passes, the user retains full control at the individual record level during manual review, where they may choose whichever version is most appropriate regardless of any default dataset previously specified. This flexibility may benefit the overall systematic review workflow, for example, by preserving the better source for a particular database combination or by aiding full-text retrieval from a preferred source.

*Text Normalization*: It enhances deduplication by standardizing textual inputs before comparison. This includes converting text to lowercase and ignoring punctuation, which mitigates variations resulting from formatting differences, typographical errors, or inconsistent data entry. By reducing discrepancies in titles and author names, normalization improves both sensitivity (detecting true duplicates) and specificity (avoiding false positives). Given these benefits, normalization is enabled by default for title-based matching in most deduplication workflows, consistent with standard practice in citation deduplication. Researchers working with datasets containing non-Latin scripts or extensively abbreviated metadata should verify that normalization performs as expected in their specific dataset before proceeding with full-scale resolution.

*Customization Protection*: It protects any articles with customized actions, such as assigned decisions, reasons, or labels, from being accidentally deleted during autoresolution (default: on). Customization Protection logic also checks if the user has taken a manual deduplication decision to keep the article. This feature is beneficial when importing new records after a review has already started.

## Discussion

### Overview

This discussion provides an overview of Rayyan’s integrated deduplication capabilities, as previously reported [10], but with the enhanced feature of the Systematic Auto Resolver to support the Rayyan Method. While several software platforms offer deduplication features, many rely on fully automated algorithms with limited user control [5,7,13]. These tools may be efficient but can lack the flexibility needed to address nuanced deduplication scenarios, such as the problem posed by dirty data, including missing, incomplete, and nonstandardized metadata. Rayyan’s Systematic Auto Resolver combines the strengths of more sensitive detection with automation and user control, allowing review teams to tailor the deduplication process to their specific needs.

### Understanding the Systematic Auto Resolver Performance in Deduplication

Unlike fully automated deduplication tools that apply fixed, predetermined algorithms without user input, the Systematic Auto Resolver executes only the criteria explicitly selected and authorized by the research team. While Rayyan provides recommended default settings to guide users toward established best practices, the criteria driving duplicate resolution are not fixed; instead, they are configurable, transparent, and documented at the user’s discretion. As a result, the Rayyan Method for Deduplication, including the Systematic Auto Resolver, reflects the stringency and appropriateness of the criteria determined by the research team rather than a black-box algorithm. This design ensures that deduplication standards align with each review’s specific requirements and that the transparent reporting of user-applied criteria provides for the auditability and reproducibility of the deduplication method used.

### Methodological Advantages

With its simple 2-stage process, the Rayyan Method can identify potential duplicates across datasets containing hundreds of thousands of references, leveraging its cloud-based infrastructure to handle large-scale work efficiently. In the detection stage, Rayyan produces a list of possible duplicates, each assigned a similarity score and presented in a clear table view, allowing researchers to assess which records may be duplicates and proceed to the resolution stage.

The resolution stage offers extensive customization, setting it apart from other tools. Researchers can view and manually review suspected duplicates, making decisions based on their specific criteria. They can also apply normalization rules, such as converting text to lowercase or omitting punctuation, to improve matching accuracy. This is particularly valuable when consolidating references exported from multiple databases, where differences in formatting conventions (eg, title capitalization, punctuation style, or author name formatting) can cause true duplicates to appear as distinct records. By applying normalization rules prior to comparison, researchers reduce the likelihood that format-driven inconsistencies across sources will result in missed duplicates or inflated reference counts.

Additionally, users can consider multiplicity by examining publication types or correcting previous record discrepancies. Rayyan’s ability to allow for the combination of systematic rule application and manual review enhances accuracy while avoiding the cumbersome, rigid workflows seen in some manual methods or the concerns of false positives of other automated methods. Research teams may apply criteria sequentially, reviewing results after each pass. This allows teams to begin with conservative rules, observe their impact on resolving possible duplicates, and then decide whether to proceed with manual resolution or adjust to a different set of criteria. Ultimately, this approach maximizes both performance and convenience, accommodating the unique characteristics of each dataset and ensuring transparent, reproducible results while preserving ultimate decision-making for the reviewer.

### Transparency and Auditability

This tutorial intends not only to provide a step-by-step guide to the enhanced deduplication process in Rayyan but also to highlight the benefits of this feature to review teams. It is important to distinguish between the two stages of the Rayyan Method: duplicate detection, which uses an algorithm to identify and score possible duplicates, and duplicate resolution, which is entirely determined by the end user. While the detection algorithm automates the identification of candidate duplicates, all resolution decisions, including the criteria applied and the final determination for each record, are made exclusively through human control and oversight. This design does not eliminate automation from the deduplication process but ensures that automation is confined to candidate identification rather than final decision-making.

This distinction is critically impactful to 2 overarching objectives of the Rayyan platform: (1) reducing the risk of introducing bias through opaque automated resolution decisions, what we term vendor bias, while acknowledging that user-controlled resolution is only as rigorous as the methodological competency of the research team applying it and (2) providing a reproducible, auditable process that upholds the foundation of systematic review methodology. Transparency and auditability are critical in systematic reviews, particularly in regulatory contexts. Rayyan facilitates this by providing a clear record of all resolution decisions, including the user-specified criteria applied to the Systematic Auto Resolver and the rationale for resolving individual duplicates. This level of documentation is intended to enhance the credibility and defensibility of the review’s methodological process.

### Limitations and Considerations

While the Rayyan Method provides significant control, this flexibility requires careful consideration by users. Incorrectly configured criteria could result in either false negatives (failing to identify true duplicates) or false positives (incorrectly identifying distinct records as duplicates), limited to the detected candidates. For example, deduplicating by DOI alone is theoretically feasible given the reasons for DOIs in the first place. However, the amount of erroneous, missing, and shared DOIs increases the possibility that this singular deduplication criterion alone can lead to false positives or false negatives. Adding more criteria increases accuracy, while reducing criteria increases sensitivity. Rayyan is under continuous development. Future development of the Systematic Auto Resolver is expected to continuously improve the accuracy, sensitivity, and usability of this capability across diverse use cases. Feedback from the research community serves as a major source of inspiration to address real-world contexts and identify the types of duplicates that are most challenging to resolve.

## Conclusion

Rayyan’s duplicate detection and Systematic Auto Resolver features offer a unique combination of user control, flexibility, and support for transparent workflows. By empowering review teams to tailor the deduplication process to their specific needs, Rayyan contributes to more accurate, reproducible, and efficient systematic reviews. While this manuscript provides a descriptive overview of Rayyan’s deduplication features, further empirical research will help guide future development. We encourage review teams to leverage Rayyan’s deduplication features to enhance the rigor and efficiency of their systematic reviews while also contributing to the growing body of evidence on the effectiveness of different deduplication methods.

## Abbreviations

PRISMA: Preferred Reporting Items for Systematic Reviews and Meta-Analyses

## References

[1] Page MJ, McKenzie JE, Bossuyt PM (2021). The PRISMA 2020 statement: an updated guideline for reporting systematic reviews. BMJ.

[2] Whitlock EP, Lopez SA, Chang S, Helfand M, Eder M, Identifying FN (2008). Methods Guide for Effectiveness and Comparative Effectiveness Reviews.

[3] (2008). Methods Guide for Effectiveness and Comparative Effectiveness Reviews.

[4] Bramer WM, Giustini D, de Jonge GB, Holland L, Bekhuis T (2016). De-duplication of database search results for systematic reviews in EndNote. J Med Libr Assoc.

[5] Hair K, Bahor Z, Macleod M, Liao J, Sena ES (2023). The Automated Systematic Search Deduplicator (ASySD): a rapid, open-source, interoperable tool to remove duplicate citations in biomedical systematic reviews. BMC Biol.

[6] Forbes C, Greenwood H, Carter M, Clark J (2024). Automation of duplicate record detection for systematic reviews: deduplicator. Syst Rev.

[7] McKeown S, Mir ZM (2021). Considerations for conducting systematic reviews: evaluating the performance of different methods for de-duplicating references. Syst Rev.

[8] Rathbone J, Carter M, Hoffmann T, Glasziou P (2015). Better duplicate detection for systematic reviewers: evaluation of Systematic Review Assistant-Deduplication Module. Syst Rev.

[9] Typology of duplicate records in systematic review context. Medium.

[10] Ouzzani M, Hammady H, Fedorowicz Z, Elmagarmid A (2016). Rayyan-a web and mobile app for systematic reviews. Syst Rev.

[11] Mosqueira-Rey E, Hernández-Pereira E, Alonso-Ríos D, Bobes-Bascarán J, Fernández-Leal Á (2023). Human-in-the-loop machine learning: a state of the art. Artif Intell Rev.

[12] Scott AM, Forbes C, Clark J, Carter M, Glasziou P, Munn Z (2021). Systematic review automation tools improve efficiency but lack of knowledge impedes their adoption: a survey. J Clin Epidemiol.

[13] Guimarães NS, Ferreira AJF, Ribeiro Silva RC (2022). Deduplicating records in systematic reviews: there are free, accurate automated ways to do so. J Clin Epidemiol.

[14] Janka H, Metzendorf MI (2024). High precision but variable recall – comparing the performance of five deduplication tools. J Eur Assoc Health Info Libr.

[15] Hammer B, Virgili E, Bilotta F (2023). Evidence-based literature review: de-duplication a cornerstone for quality. World J Methodol.

